# Investigating the Effects of Cement and Polymer Grouting on the Shear Behavior of Rock Joints

**DOI:** 10.3390/polym14061229

**Published:** 2022-03-18

**Authors:** Milad Abolfazli, Milad Bazli, Hossein Heydari, Ahmad Fahimifar

**Affiliations:** 1 College of Engineering, IT and Environment, Charles Darwin University, Darwin 0815, Australia; 2 School of Mechanical and Mining Engineering, The University of Queensland, Brisbane 4072, Australia; 3 Department of Engineering, Kharazmi University, Karaj 15719-14911, Iran; hossain.heydari@khu.ac.ir; 4 Department of Civil and Environmental Engineering, Amirkabir University of Technology, Tehran 15875-4413, Iran; fahim@aut.ac.ir

**Keywords:** rock joint, cement grouting, chemical grouting, shear behavior

## Abstract

This study carried out a comparison between cement grouting and chemical grouting, using epoxy and polyurethane, with respect to their effects on the shear behavior of joints. Joint replicas, with three different grades of surface roughness, were molded and grouted by means of cement and epoxy grouts of various mixtures. To investigate their shear behavior, samples were subjected to direct shear tests under constant normal load (CNL) condition. According to the results obtained, grouting improves the overall shear strength of the rock joints. All the grouted samples yielded higher maximum and residual shear strength in comparison with the non-grouted joint. Grouting resulted in an improvement in the cohesion of all the samples. However, a fall in friction angle by 5.26° in the sample with JRC of nine was observed, yet it was reduced by 2.36° and 3.26° for joints with JRC of 14 and 19, respectively. Cement grouts were found to have a more brittle behavior, whereas the chemical grouts were more ductile. Higher amounts of cement used in the grout mixture do not provide as much cohesion and only increase the brittleness of the grout. As a result of being more brittle, cement grout breaks into small pieces and joint planes are in better contact during shearing; consequently, there would be less of a fall in friction angle as opposed to epoxy grout whose ductile characteristic prevents grout chipping; therefore, joint planes are not in contact and a greater fall in the friction angle occurs. There was no noticeable change in the cohesion of the larger grouted joints. However, the friction angle of both natural and grouted joints increased in the larger joint. This can be related to the distribution of random peaks and valleys on the joint surface, which increases with the joint size.

## 1. Introduction

Grouting has been one of the prominent solutions of engineers for dealing with a variety of geotechnical dilemmas, from the improvement of strength, density and porosity characteristics in loose soils, to filling the joints and cracks existing in rock and concrete [1,2,3,4,5,6]. The injection has proven its practicality and effectiveness when other approaches fail to function as a permanent and stable remedy for such problems [7]. Having high mechanical properties when completely cured as well as low viscosity, which enables it to penetrate into the finest seams, cracks, and pores in soils and fill them homogeneously, along with undisputed adhesion capability, makes grouting a unique strategy to increase the performance of geotechnical structures [8,9,10,11]. Cement has been used as the base material in grout mixtures for a long time due to ease in accessibility and price efficiency in addition to its convenient post-curing strength [12,13,14,15,16,17,18]. However, despite the beneficial traits cement-based grouts provide, there are a few considerable drawbacks that do not allow cement grouts to be applied in every situation. For instance, cement grouts could not be used when extremely fine soils like silts are to be treated since cement particles may not infiltrate the soil fine pores or while inclined cracks or joints in rock or concrete need to be filled, though gravity would force the mixture to creep out [10,15,19,20]. Chemical compounds have also been employed for grouting purposes to overcome the defects experienced with cement [19,21]. A wide range of chemical materials is used in grouting based on the ground properties and other technical considerations of the site, the most common of which include: polyurethane, sodium silicate, acrylate, and epoxy resins [10,21]. Epoxy resins are popular among the chemical solutions used for grouting due to their fast hardening duration, high viscosity, and significant strength properties when cured [22,23,24]. Similar to other chemical compounds, resins must be mixed with another component for the curing process to occur, which is known as the hardener solution [19]. Aside from some practical advantages found in epoxy resins, their high price remains an issue engineers need to face when epoxy resins are used. Epoxy resins are not often soluble in water; yet some may be diluted with water to some extent to match the viscosity requirement of the project or to reduce the cost of the grouting procedure. However, the dilution of epoxies with a higher amount of water mostly results in reduced strength and durability of the cured epoxy [12,25,26]. In rock mechanics, and especially in tunneling practices, grouting is utilized to enhance strength and deformability features of the rock mass by filling cracks and joints that may endanger stability and usability of the underground structures or rock slopes [3,27,28]. The efficiency of the grouting material is reliant on the physical features of the joints such as roughness, opening width, and the direction, along which the joint is displaced during the shearing process. To eliminate the role of joint physical features in the experiments and focus on the effects of grouting on the shear behavior of joints, several early studies suggested the production of joint replicas with natural or artificial roughness from gypsum or concrete [21,29,30]. However, gypsum does not offer appropriate strength as high as that of a rock joint, and fabrication of replicas from concrete is very time-consuming and does not provide a precise duplicate of the joint roughness [30]. Therefore, dental plaster, as a material that has nearly as high a compressive strength as rock and a curing period tantamount to that of gypsum has been used as an alternative for capturing rock joints roughness. A comparison between cement grouting and epoxy resin grouting, with respect to its effects on shear behavior of joints, was carried out in this study. As the water to cement ratio has an unambiguous impact on the final strength of the grout [1,2], two different mixtures of cement grouts and specified ratios of chemicals in the other grouts were chosen to be employed in the experiment. After several tensile fractured joint replicas were made from dental plaster, they were grouted, and their shear characteristic was investigated by performing direct shear tests in constant normal load (CNL) condition. Eventually, test results and discussion are presented to help engineers make a wise decision on grouting material choice based on the conclusions drawn from the study.

## 2. Experimental Program

Mechanical properties of rock joints are highly contingent upon the physical characteristics of the joint surface [31,32]. The presence of various factors and parameters influencing obtained results in every experiment contributes to not only an unreliable comparison but also hinders the utilization of natural rock joints in the testing procedure due to the distinctive and unique nature of such joints [33,34,35]. Thus, the only practical solution would be to fabricate physical features of rock joints i.e., roughness, size, etc. using a single material whose mechanical traits, including compressive strength, hardness, etc., could be analogous to that of rock. To peruse such an aim, tensile fractured rock joint with a variety of roughness were employed to be included in the fabrication process so the representation of the joints can be flawlessly conduced. Strength features of the re-generated grouted joints including shear strength, cohesion, and friction angle were then assessed through direct shear tests accordingly. As previously mentioned, attributes of grouting compounds were chosen with the likelihood of their applicability with engineering practices in mind.

### 2.1. Joint Fabrication Procedure

**Figure 1:**
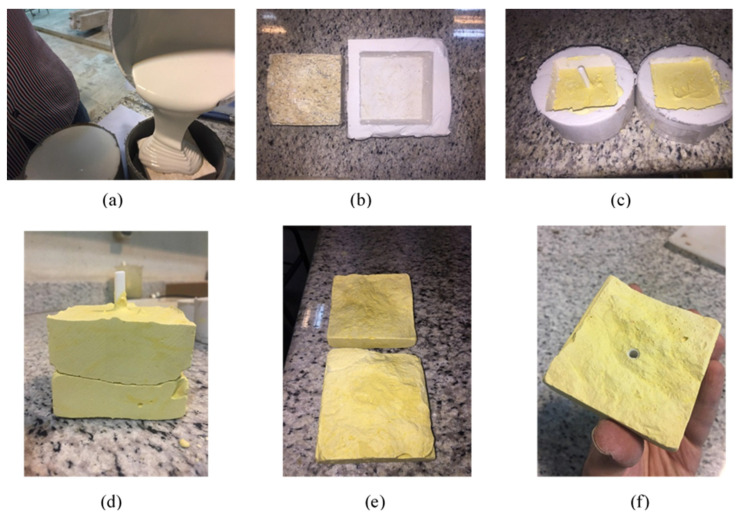
Fabricating rock joint replicas in this study: (**a**) Molding process; (**b**) Comparison of the mold produced and the rock joint; (**c**) Casting dental plaster replicas; (**d**) Complete matching in joint planes; (**e**) Surface roughness captured; (**f**) Placement of the pipe in the samples for grouting procedure.

**Figure 2:**
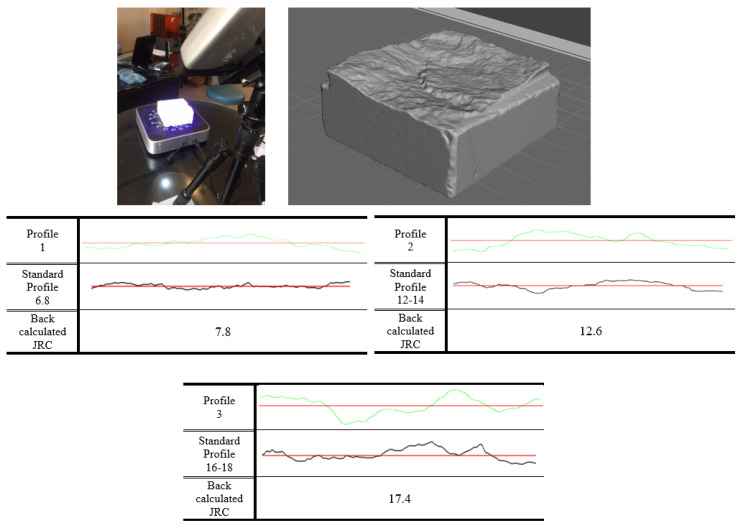
Digitizing replicas’ surface profiles and determining their JRC through visual comparison with Barton’s standard profiles and back-calculation after direct shear testing on non—grouted joints.

The rock joints used in this study were produced through fracturing cubic blocks, resulting in the generation of a tensile joint approximately in the middle of each block. Specimen blocks were sourced from travertine and granite mines located in an industrial region in the south of Tehran, Iran. The selection of these rocks was made with a view to having a variety of textures as travertine, being a sedimentary rock, features a porous and streaky formation, whereas igneous granite has a homogeneous structure; and it is known that the rock type material has a determinative role in the creation of rock joints [33,36]. Based on the roughness of the acquired produced tensile fractured joints, four distinct joint types with different joint roughness values (JRC) were selected to be cast with high precision silicon rubbers. After casting, it was required to fabricate the joints using a material that shared almost the same mechanical and physical characteristics of rocks. Furthermore, due to the necessity to generate a considerable number of specimens to be employed in the testing procedure, the chosen material was intended not to take too long to cure. Concrete, as the primary considered material, lacks fast curing trait since it usually takes at least 24 h for a specimen’s initial setting and cast removal, and 28 days to obtain final strength, despite the adequate strength characteristics. Gypsum, however, unlike concrete does not suffer from a long curing duration; nevertheless, the inadequacy of final strength as well as its brittleness prevents gypsum from serving as an appropriate material for producing artificial joints [30]. After the examination of a few other materials, dental plaster was found to be a decent candidate to serve the aims of this research. Unlike both concrete and gypsum, dental plaster enjoys high final strength when completely cured, which approximately takes seven days, and an impressively short duration of cast removal of 10 min. Some of the properties of the plaster used in this study can be seen in Table 1. Prior to engaging the material in specimen production, a uniaxial compression test was performed on cylindrical samples of dental plaster, and its strength was recorded at 51 MPa; around the same strength observed in relatively soft rocks. Figure 1 illustrates the process of casting the specimens and fabrication of artificial joints with a similar JRC profile. To determine the JRC value of each sample, 3D scans of individual specimen groups were conducted, the results of which are shown in Figure 2. In furtherance of capturing detailed roughness of the joint planes, as can be seen, respective sides of each specimen were carefully scanned using a high-resolution 3D laser scanner with a data acquisition resolution of 50 square microns. Next, the 3D data were processed with a computer, and sections through each specimen, containing the desired profiles, along which later direct shear tests were planned to be conducted, were made. A sampling interval of 0.02 mm was chosen to extract the surface profile data from the scanned data file. The scan data were later used to evaluate the precise JRC value of the joint via several statistical correlations [37,38], as well as a visual comparison to Barton’s [39] ten standard profiles. Finally, the JRC obtained for the three groups of specimens were 7.8, 12.6 and 17.4.

### 2.2. Grouting

As it was highlighted earlier, grouting is exploited in diverse geotechnical engineering practices, nevertheless, in rock engineering, it is simply used to improve the mechanical and hydraulic behavior of a rock mass by adhering blocks separated with intersected joints or filling up interconnected conduit-like joints to stop water-stream from flowing into an underground space [10,21,40]. The solution to be employed in the grouting operation must fulfill certain requirements such as high tensile and shear strength, adequate viscosity, short initial setting duration together with maintaining proper additives to help with the durability if the highest safety and functionality of the structure is a circumstance of concern [12,19,41,42]. Cement has long been used in grouting due to its impressive strength and competitive cost in comparison with other substances. Recently conducted research on the effects of different properties of the cement-based grouting solutions, including water to cement ratio, cement type, grain size, and various additives, showed how profound the choice of grouting mixture can be [9,14,30]. On the other hand, there are a few chemical compounds like epoxy resins whose traits have made them worthy of being used as a grouting agent, and despite their relatively steep price, some of their upsides like the fast curing duration and very high strength properties turn them into an advantageous replacement for cement in the grouting operation [10]. Costing impediments of epoxies can be avoided should another chemical solution be proposed with similar benefits to the epoxy grouts. The appropriacy of using chemical-based grouting solutions, in comparison with those of cement-based, and investigating the cost and strength efficient water to the proxy ratio has received little attention; thus, they were selected as the main objective of the present research.

The cement used in this study was Portland cement type 2, which is broadly used in geotechnical soil and rock mass improvement practices. The epoxy resin employed in the current research is manufactured by Sika Co. with the trading name Sikadur 52. It is a solvent-free, 2-component epoxy resin that is highly impermeable and produces fantastic tensile strength at nearly 37 MPa after curing for seven days (Maximum strength is achieved when no water is added to the epoxy). Polyurethane used in this study was manufactured by Sika Co. with the trading name “SikaBond”. To investigate the effects of grout mixture on shear behavior of joints, different mixtures for each grout type were utilized, and these properties are given in Table 2.

In order to inject the grout into the joints, a pneumatic pipe six mm in diameter was placed in the dental plaster paste, while molding each specimen and the grout was injected through the pipe at 1.5 bar pressure. This pressure was chosen so that complete and consistent penetration of the grout into the joint was ensured. During the injection procedure, all four sides of the specimen where the joint was located were taped to prevent excessive grout run off; in the meantime, specimen halves were secured tightly together to prevent considerable normal deformation of the joint. Those specimens injected with cement-based grouts were kept submerged for 28 days so the cement would attain its final strength, whereas epoxy and polyurethane grouted specimens were left in the room environment for seven days to be cured completely.

### 2.3. Direct Shear Testing

**Figure 3:**
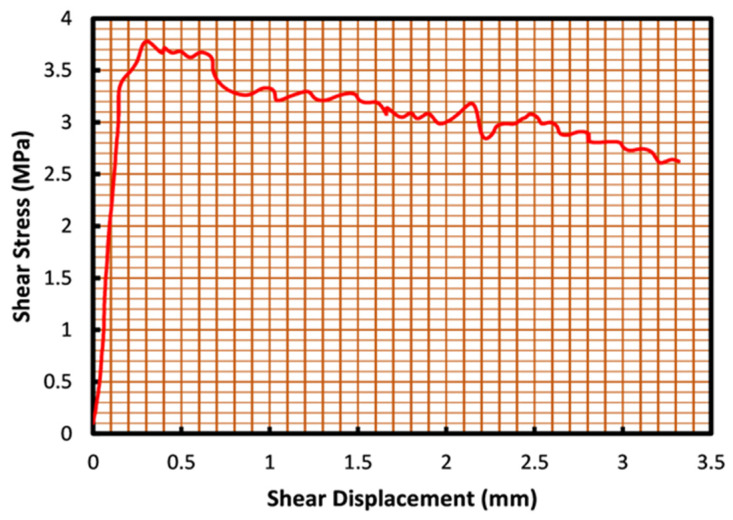
Example of the stress-displacement curve for an epoxy grouted joint with JRC of 17.4 derived through a direct shear test.

**Figure 4:**
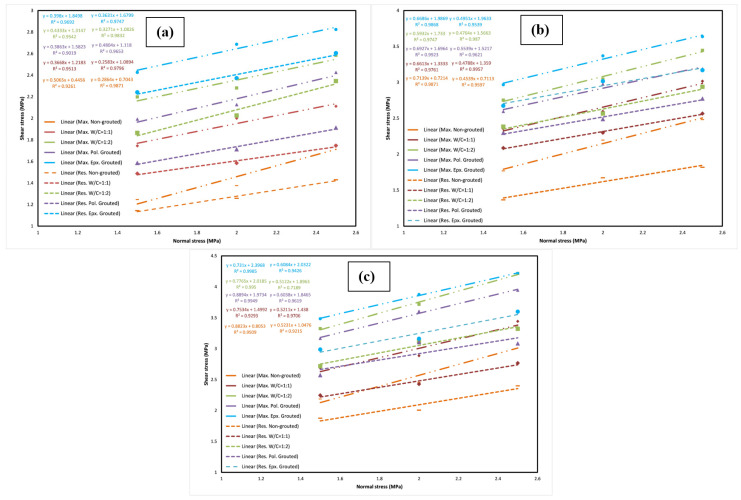
Shear stress-normal stress curves obtained for rock joints for (**a**) JRC = 7.8, (**b**) JRC = 12.6, (**c**) JRC = 17.4.

The direct shear testing procedure in the present research was performed at constant normal load condition (CNL), which means that the normal load on specimens was kept constant throughout the shearing. The rock direct shear instrument was utilized in this study, which employed hydraulic pumps as a load exertion system with a loading capacity of 10 KN. Cement-based grouted joint specimens were tested after 28 days of curing, whereas epoxy-based grouted joints had undergone shearing seven days following grout injection. During the shearing procedure, normal and shear displacements were measured using five linear variable differential transformers (LVDT) and a load cell was responsible for recording variations in shearing load throughout the test. Stress-displacement curves were produced after every single test, from which shear strength and residual strength of the grouted joint were derived (Figure 3). Shear stress-normal stress curves were then obtained through multiple testing at different normal stress levels (Figure 4). As can be observed, load-displacement curves of the joints demonstrate the typical ascending trend at low shear displacement and following a peak, which is referred to as the joint shear strength, a fall in strength precedes the relatively constant residual strength of the grouted joints. Shear stress-normal stress plots have an ascending trend, from which, considering the Mohr–Coulomb criteria, joints cohesion, and friction angle were determined and compared. Joint samples were scanned prior and post shearing using a 3D laser scanner to keep records of surface roughness changes. Three surface profiles of each sample were extracted from the 3D data and compared against Barton standard profiles to provide the mean JRC of each joint. Samples were tested with three different roughness coefficients, grouted with four different grouting solutions tested at three different normal stress levels; in addition to the nine tests on non-grouted joints that were also conducted to measure their shear strength. Furthermore, fifteen tests were conducted to inspect the effect of joint size on the shear behavior of the rock joints. An overall number of 60 tests were conducted in this study.

## 3. Results and Discussions

JRC values for the profiles were determined through back-calculation of Barton as follows:

(1) $$\tau =\sigma_{n}\tan\left(\mathrm{JRC}\log\left(\frac{\mathrm{JCS}}{\sigma_{n}}\right)+\phi_{b}\right)$$

where JRC and JCS are joint roughness coefficient and joint compressive strength, respectively, and $\phi_{b}$ is the basic friction angle of the rock joint. Separate direct shear tests under constant normal load condition at 1 MPa of normal stress were performed on none of the grouted joints and a JCS value was derived from Equation (2):

(2) log(JCS)=0.00088rR+1.01(MPa)

where r represents rock density and *R* is the dimensionless rebound factor using the Schmitt hammer. $\phi_{b}$ was calculated based on similar shear tests performed on completely smooth surface specimens. Three shear tests are performed and the slope of the line connecting the three results in a $\tau -\sigma_{n}$ chart would be $\phi_{b}$.

The normal stress applied on the shear zone of each specimen is recognized by Equation (3):

(3) σn=NA

where $\sigma_{n}$ is constant normal stress, *N* denotes the constant normal load imposed by the machine and A is nominal area of the joint shear zone. It is worth mentioning that the weight of the upper specimen holder is accounted for when adjusting the intended normal load on the machine such that no extra stress, aside from the desired amount, is applied.

The max shear stress applied is recognized by Equation (4):

(4) τmax=VmaxA

where Vmax is the maximum load recorded by the load cell during the direct shear test and A is the nominal area of the joint shear zone. As the shear load during the test is changing, only the maximum value is considered as the shear strength.

### 3.1. Effects of Grouting on Shear Strength of Rock Joints

An investigation was carried out to determine the effects of grouting on the shear strength of rock joints. The principal parameters include maximum and residual shear strength, friction angle and cohesion, and peak shear stress displacement. Joint normality and shear stiffness were also inspected.

#### 3.1.1. Shear Strength vs. Normal Stress Curves

Shear strength against normal stress plots regarding each group of test samples are demonstrated in Figure 4. Samples are nominated in one of the four groups based on their surface roughness. A linear regression of the three tests of each sample was performed for both peak and residual shear strengths whose slopes correspond to the joint friction angle with Y-intercept representing cohesion. As can be seen in Figure 4a, for the joint with JRC = 7.8, cohesion at the maximum shear stress has increased by 173.41% for the w/c ratio of 1:1 and 255.10% for the w/c ratio of 1:2 in comparison to the non-grouted joint. Moreover, it can be observed that the same parameter has increased by 195.04% and 314.95% for the polyurethane grouted and epoxy grouted joints, respectively. In addition, at the residual shear stress, cohesion increased by 54.68% and 32.23% for the specimens with w/c ratio of 1:1 and 1:2, and it was escalated by 53.71% and 138.52% for the polyurethane and epoxy grouted joints, respectively. The friction angle represented by the slope of the regression lines was evidently decreased at maximum shear stress following grouting regardless of the grout type. According to Figure 4a, the fall in curve slopes for joints with w/c ratios of 1:1 and 1:2 at maximum shear stress are 27.58% and 23.73%. In epoxy grouted specimens also, the reduction in the slope at maximum shear stress is 14.45% for polyurethane grouted joints and 21.42% for epoxy grouted joints. This means that in cement grouted joints, a higher amount of cement causes less of a drop in the friction angle, yet in epoxy grouted joints a higher epoxy in the grout mixture leads to more of a drop in the friction angle. Figure 4b depicts the curves regarding the joint with JRC = 12.6. As can be seen, cohesion at the maximum stress is increased by 84.82% for the w/c ratio of 1:1 and 135.15% for the water to cement ratio of 1:2, while for the epoxy grouted joint it is 140.23% and 175.72% for polyurethane and epoxy grouted joints, respectively, comparing to the non-grouted joint. In terms of cohesion at residual shear stress, there is a rise of as much as 91.06% and 113.93% for w/c ratio of 1:1 and 1:2. Additionally, cohesion at residual shear stress for epoxy grouted specimens increased by 120.20% and 176.01% for polyurethane grouted joints and epoxy grouted joints, respectively. It can be inferred from Figure 4b that the line slope changes show a 7.37% and 2.97% fall in the cement grouts with w/c ratios of 1:1 and 1:2. The reduction in slope is 16.91% and 6.34% in the case of the epoxy grouts with e/w ratios of 1:1 and 1:2. Grouting seems to have reduced the lines’ slope in all specimens at maximum shear stress, yet at residual stress a slight boost in the friction angle is observed. Figure 4c demonstrates corresponding curves to fabricated joints with JRC = 17.4. As can be seen, for cement grouted joints with w/c ratio of 1:1 and 1:2, cohesion at maximum shear stress has increased by 80.17% and 145.05%. However, this parameter seems to have risen by 150.65% and 197.62% for polyurethane and epoxy grouted joints, respectively. Furthermore, cohesion at residual shear stress escalated by 37.27% and 76.26% for w/c ratios of 1:1 and 1:2. In addition, it spiked by 81.01% and 93.99% for polyurethane and epoxy grouted joints. Similar to what was observed with the joint with JRC = 12.6, a fall in the friction angle at maximum stress following grouting is seen in most specimens. Figure 4 represents curves regarding the 11 cm joint, which has the same joint roughness coefficient (JRC) as the first set of specimens (JRC = 7.8). Cohesion at maximum shear stress was increased by 33.25% and 85.92% for cement-grouted specimens with w/c ratio of 1:1 and 1:2. Furthermore, for epoxy-grouted specimens the same parameter was boosted by 63.79% and 104.06% for polyurethane and epoxy grouted joints, respectively. Cohesion at residual shear stress was also increased. For w/c ratios of 1:1 and 1:2 it escalated by 15.92% and 59.65%. Moreover, it rose by 36.58% and 95.13% for polyurethane and epoxy grouted joints. The friction angle was increased in comparison to the smaller joint. However, a drop in the lines’ slope was observed in all grouted samples.

#### 3.1.2. Normal Stiffness and Shear Stiffness of the Grouted Rock Joints

**Figure 5:**
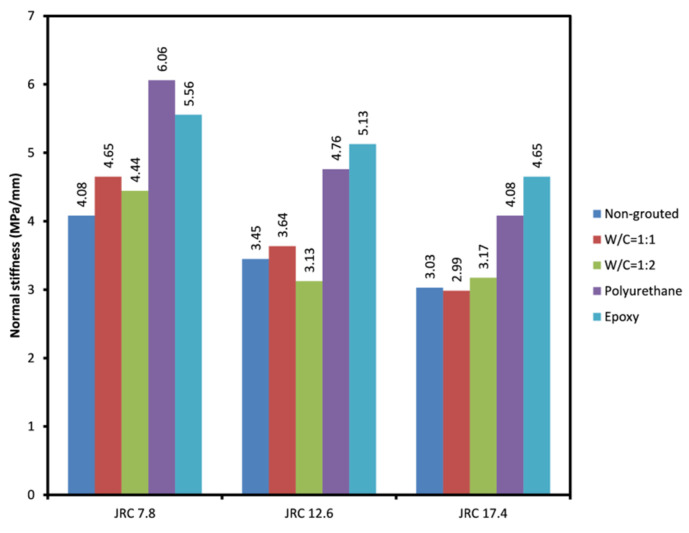
Average normal stiffness obtained for the rock joints tested.

**Figure 6:**
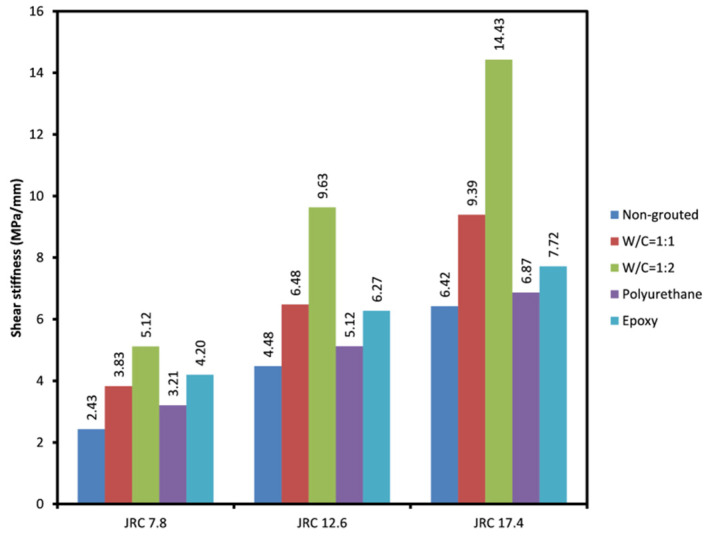
Average shear stiffness obtained for the rock joints tested.

Figure 5 and Figure 6 depict the average maximum normal stiffness and shear stiffness of different grouted joints tested. As can be seen, grouted joints show a more normal stiffness in comparison to non-grouted joints. This can be due to the fact that grouting, as shown before, has a negative impact on the friction angle of the joints resulting in less of a normal deformation, which in turn leads to a rise in normal stiffness. It is clear that a more prominent rise is observed in polyurethane and epoxy grouted joints compared to cement grouted joints. This is due to the cement grout being chipped following failure, which acts as additional roughness and leads to more normal displacement of the joint planes. Polyurethane grouted joints with JRC = 7.8 seem to have experienced a slightly greater rise in normal stiffness compared to epoxy grouted joints with similar JRC. Shear stiffness of the grouted joints was also investigated. Figure 6 shows that cement grouted joints have higher shear stiffness than epoxy and polyurethane grouted joints. This represents a more brittle behavior of cement grouts. Chemical grouts, especially epoxy grout, allow for more shear deformation prior to grout failure, and this results in a lower shear stiffness in those grouted joints. It was also understood that shear and normal stiffness of the grouted joints are highly reliant on the joint aperture and how well-mated the joints are. The higher aperture allows greater amounts of grouts in the joint, which affects the overall shear behavior of the joint to a great extent. In the present research, it was observed that joints with irregular aperture along their lengths, where a part of the joint planes were fully interlocked while other parts were not in contact, produced more normal stiffness and less shear stiffness compared to joints that have fully interlocked planes. Consequently, the rougher and better-mated joints produce less normal stiffness and more shear stiffness.

#### 3.1.3. Effect of Roughness

**Figure 7:**
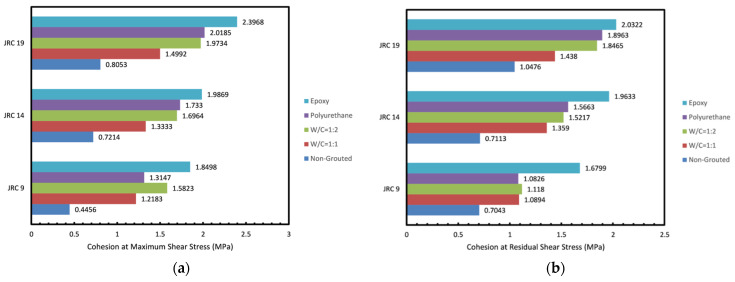
(**a**) Cohesion at maximum and (**b**) Cohesion at residual shear stress obtained for the rock joints tested.

**Figure 8:**
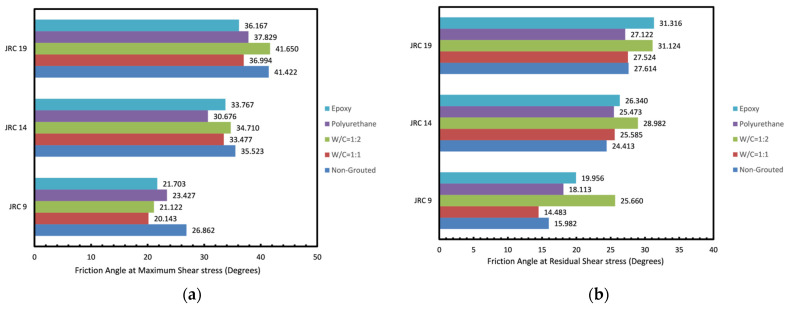
Friction angle at (**a**) maximum shear stress and (**b**) residual shear stress obtained for the rock joints tested.

In order to investigate the effect of joint roughness on the strength of grouted joints, cohesion and friction angle changes at maximum and residual stress are demonstrated in Figure 7 and Figure 8. As reported in previous studies, it can be seen that an increase in roughness pertains to a higher cohesion and friction angle. Figure 8 shows that grouting contributes to a fall in friction angle at maximum shear stress as the injected grout acts as an infill material and prevents the complete interaction of the two joint planes. The better mated and rougher rock joints were found to be less susceptible to being affected as the non-uniform accumulation of grout slurry over the joint plane was prevented. The rock joint with the lowest JRC = 9 experienced the most affected post grouting as the friction angle at maximum shear stress in all specimens was decreased by a mean of 5.26°, whereas for joints with a JRC of fourteen and nineteen, it only fell by a mean of 2.36° and 3.26°, respectively. This is in contrast to the results reported in an earlier study [30], where no specific trend in friction angle changes based upon roughness was found.

#### 3.1.4. Effect of Grout Type and Mixture Ratio

In this study, two grout types were employed and the effect on the shear strength of the joints was investigated. As mentioned in the introduction, epoxy grouts offer numerous positive characteristics to the overall shear strength and durability of the grouted joint over the conventional cement-based grouts used. According to shear stress vs. normal stress curves, one can understand that those that were epoxy-grouted yielded better strength properties in comparison to cement-grouted specimens. According to Figure 7, epoxy-grouted joints yield the highest cohesion. Moreover, polyurethane grouted joints and cement grouted joints with w/c ratio of 1:1 produce nearly similar cohesion. This proves the better efficiency of the epoxy grout over the cement grout used. It can also be inferred that higher ratios of epoxy and cement do not increase the cohesion generated as the higher ratio only adds a mean of 0.4 MPa to cohesion in cement grouted joints and a mean of 0.39 MPa in epoxy grouted joints. Results obtained in Figure 8 show that the cement-grouted specimens with w/c ratio of 1:1 experienced the lowest fall in friction angle; dropping 2.1 degrees at maximum shear stress in comparison to the non-grouted joint. However, at residual shear stress grouting mostly led to a rise in friction angle with the highest boost generated in the joint with the lowest JRC and the lowest in the joint with the highest JRC. This might be explained through the fact that the failure plain in the grout presents more roughness than the joint planes themselves. This causes a more significant difference in residual stress in joints with lower original JRC.

**Figure 9:**
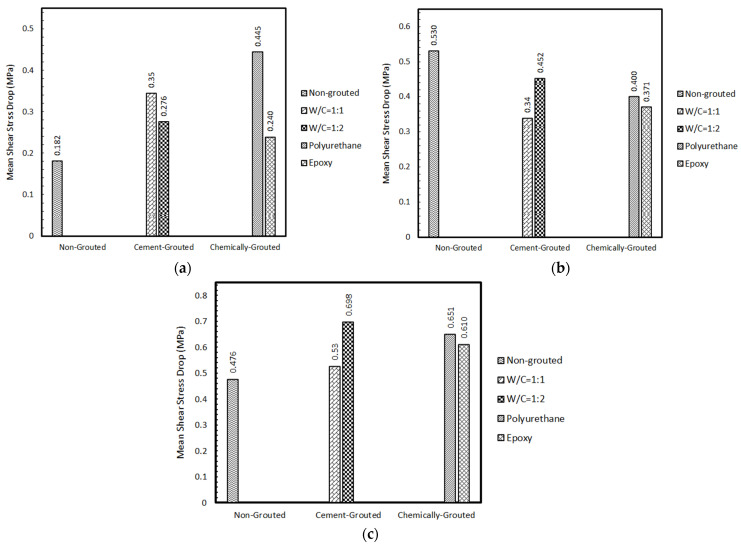
Comparison of mean shear stress drops across various joints tested. (**a**) JRC = 7.8, (**b**) JRC = 12.4, (**c**) JRC = 17.6.

**Figure 10:**
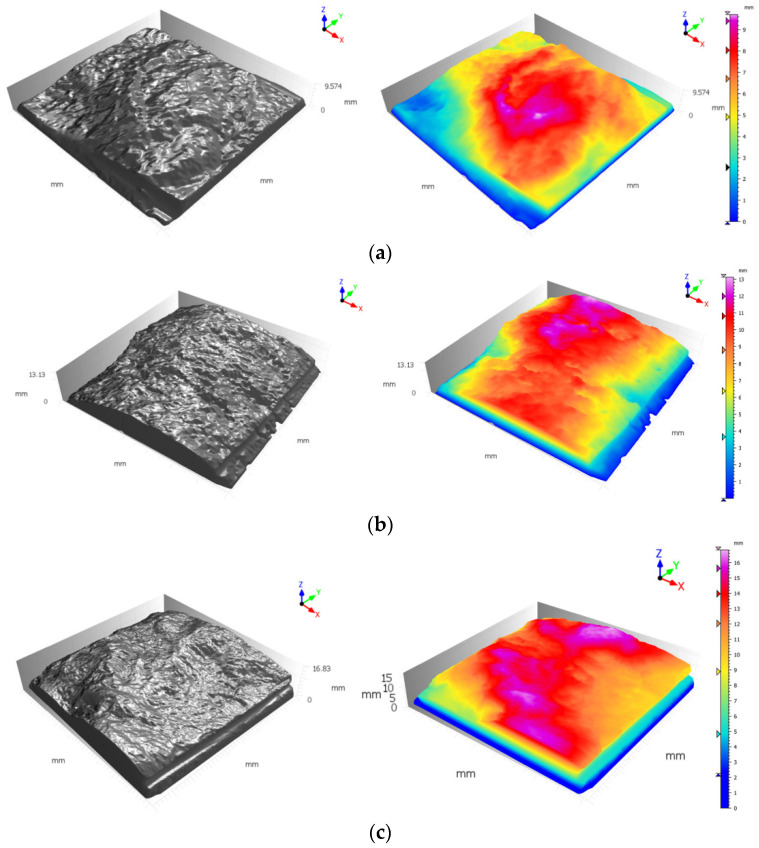
Digitized, 3D scanned of the fabricated rock joints used before the direct shear test (**a**) JRC = 7.8, (**b**) JRC = 12.4, (**c**) JRC = 17.6.

**Figure 11:**
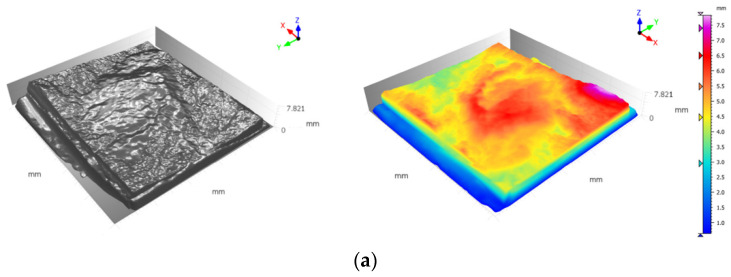

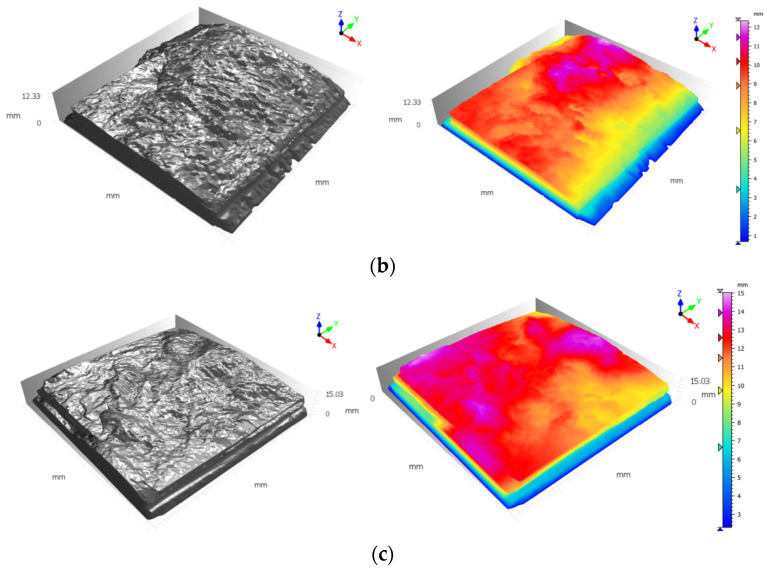
Digitized, 3D scans of the cement grouted fabricated rock joints used after direct shear test (**a**) JRC = 7.8 cement grouted w/c = 1:1, (**b**) JRC = 12.4 epoxy grouted, (**c**) JRC = 17.6 cement grouted w/c = 1:2.

Figure 9 represents the difference between maximum and residual shear stress in various grouting scenarios and different joint roughness. As can be seen in Figure 9a, grouting has resulted in an increase in shear stress drop, which can be inferred as a more brittle behavior of the joint. This could be justified in the light that at a lower JRC, joint planes would most likely slip on one another; therefore, there is not much difference between maximum and residual shear strength produced. However, following the grouting procedure, joint failure is chiefly governed by the behavior of the grout material. Hence, grouted joints with lower JRC experienced a greater drop in shear strength than the non-grouted joint. It should also be mentioned that as epoxy grout has a more ductile behavior than cement grout, epoxy-grouted joints experienced less of a fall in shear strength. This can be introduced as a negative effect of grouting as a ductile behavior is desirable due to safety standards. Therefore, when grouting joints with lower JRC, it is recommended that engineers opt for epoxy grouts that yield less of a drop in shear strength. According to Figure 9b,c, as the joint roughness increases one cannot draw a clear conclusion on the behavior of the joints as the shape and distribution of asperities can have a noticeable impact on the shear behavior of grouted joints. Nevertheless, it is still clear that the epoxy grouted specimen showed a slightly more ductile shear behavior. Figure 10 and Figure 11 depict digitized 3D presentation of joints planes before and after direct shear tests. From Figure 11 one can understand that in the cement grouted joints more damage was inflicted on the joint roughness (the asperities heights are reduced more significantly) as crashed pieces of cement are abraded between joint planes after grout failure, which resulted in a higher residual friction angle. Since these crashed pieces are smaller than the average asperities height of the joint, they contribute to a rougher surface. This effect becomes more prominent as the asperities height rise (higher JRC), leading to a less normal stiffness as seen in Figure 9. It can be observed in Figure 11 that the joint with JRC of 7.8 and 17.4 grouted using cement with w/c ratio of 1:1 and 1:2, respectively, have experienced a larger fall in asperities height of nearly 2 mm. However, epoxy and polyurethane grouted joints were damaged less as the chemical grout acts as an anti-erosion thin layer on the asperities, keeping them from abrasion. Consequently, these joints suffered less of a drop in asperities height due to the shear. As seen in Figure 11, the rock joint grouted using epoxy has experienced a minor drop in asperities height of 0.8 mm following the shear test.

On the other hand, cement and epoxy grouts seem to have filtered through the joints better while the polyurethane grout failed to deliver such infiltrative properties, leaving some areas of the joint non-grouted. Thus, using a less viscous chemical solution can facilitate grout dispersion on the joint surface and higher grout strength.

#### 3.1.5. Effect of Joint Size

**Figure 12:**
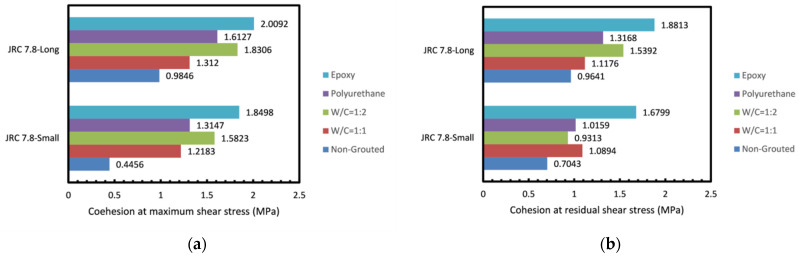

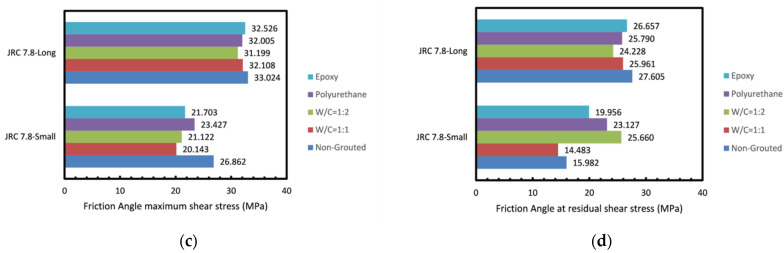
Comparison of the friction angle and cohesion of small and long rock joints tested. (**a**,**b**) cohesion at maximum and residual shear stress, (**c**,**d**) friction angle at maximum and residual shear stress.

There has been extensive research on the effect of joint size on the shear behavior of rock joints. According to these studies, both positive and negative effects of size on shear behavior of joints could be expected [43,44]. Based on the pertinent research, a plethora of parameters might have an impact on shear behavior of joint and size cannot be solely accounted for the positive or negative effect. As can be seen in Figure 12, a positive effect of size on both cohesion and friction angle of the tested joints was observed. This can be attributed to the random distribution of asperities over the joints, which becomes more prominent as the joint gets greater in size. In agreement with a number of previous studies, a high degree of interlocking and well-mated surface joints in the present samples are responsible for the positive size effect. It can be observed that grouting by means of cement grout with w/c ratio of 1:1 and epoxy grout yielded the highest cohesion. It is also clear that as the specimen was enlarged, no noticeable rise in cohesion after grouting occurred. In addition, no trend through which cohesion changes could be described was found. Friction angle of joints post grouting was decreased.

## 4. Conclusions

This study was carried out to investigate the effects of grouting on shear behavior of artificially made rock joints. In doing so, a number of rock joint replicas were made using dental plaster, which provides similar strength as rock and possesses great molding property, as well as precise surface roughness production. Next, specimens were grouted with various cements and epoxy grout mixtures and then underwent direct shear tests.

According to the results obtained, grouting improves overall shear strength of the rock joints. All the grouted samples yielded higher maximum and residual shear strength in comparison with the non-grouted joint. Roughness was found to be a significant factor affecting shear behavior of the joints. As the roughness of the sample increased, cohesion and friction angle of the natural joint increased. However, grouting resulted in a fall in friction angle. The reduction in friction angle post grouting was more prominent in samples with lower JRC. This was ascribed to excessive accumulation of the grout slurry on some parts of the joint surface where two planes were not in perfect contact. As a result, a fall in friction angle of 5.26° in the sample with JRC of 7.8 was observed, yet it was reduced by 2.36° and 3.26° for joints with JRC of 12.6 and 17.4, respectively. Two different grout types were used in this study. Cement grouts were found to have a more brittle behavior whereas epoxy grouts were more ductile. Polyurethane grouted joints yielded nearly the same amount of cohesion as cement grouted joints with w/c ratio of 1:1, which implies great efficiency of polyurethane grouts. It was also observed that higher amounts of cement used in the grout mixture do not provide as much cohesion and only increase brittleness of the grout. It was also understood that there is a greater difference between maximum and residual shear stress following grouting, which can be interpreted as a more brittle behavior of rock joints. As a result of being more brittle, cement grout, breaks into small pieces and joint planes are in better contact during shearing; consequently, there would be less of a fall in friction angle as opposed to epoxy grout, which has a ductile characteristic that prevents grout chipping; therefore, joint planes are not in contact and a greater fall in friction angle occurs. The rougher the surface becomes, the less its friction angles fall due to grouting. It should also be mentioned that polyurethane grout lacked infiltrative characteristics in comparison to cement and epoxy grouts, which in turn left a few spots non-grouted.

Previous research carried out on cement grouted joints have shown similar trends found in the present study. M.H. Salimian et al. [30] have reported a 5–48% fall of maximum friction angle as well as a 59–282% boost of maximum cohesion in joints grouted at w/c ratios of 1:1 and 2:1. The present research also demonstrates a drop of 3–28% in maximum friction angle and an 84–197% rise of maximum cohesion across all samples tested. As mentioned earlier, the shear behavior of grouted rock joints has not been studied rigorously so the results obtained in this study could be used as a ground for extensive research in this matter.

The joint size was found to have a positive effect on the shear behavior of joints. As a number of previous studies suggest, a well-mated and high degree of interlocking can be responsible for the positive size effect. There was no noticeable change in cohesion of the enlarged grouted joint. However, the friction angle of both natural and grouted joints increased in the larger joint. This can be related to the distribution of random peaks and valleys on the joint surface, which increases with the joint size.

## Figures and Tables

**Table 1:** Properties of the dental plaster used in this study.

	Density (kg/m^3^)	W/P * Ratio	Uniaxial Compressive Strength (N/mm^2^)	Elastic Modulus (N/mm^2^)	Setting Time (Mins)	Expansion Ratio (%)
Dental plaster	2630	0.25	48.6	29,400	15	0.05–0.25

* W/P Represents the water to powder ratio of the plaster mixed.

**Table 2:** Properties of the grouts used in this study.

Grout Type	“w/c” or “A/B *” Ratio	Density (kg/m^3^)	Tensile Strength (N/mm^2^)	Internal Friction Angle (Degrees)	Cohesion (N/mm^2^)	Elastic Modulus (N/mm^2^)	Viscosity (at 30°-mPa·s)
Cement	1:1	1492	2.4	38.76	4.23	17,600	580
Cement	1:2	1750	2.7	53.24	7.49	18,500	730
Epoxy	2:1	1100	37	13.66	25.73	1800	220
Polyurethane	-	1110	3	11.49	8.12	1630	4000

* A/B Represents the mixture ratio of components A and B.

## Data Availability

Not applicable.
